# Exploring Maternal and Infant Health App Development and Effectiveness Research: Scoping Review

**DOI:** 10.2196/46973

**Published:** 2024-01-26

**Authors:** J Craig Phillips, Alliete R Alfano, Latisha C Barfield, Lisa Cain, Masoud Sadjadi, Eduardo Morales, Wanda Phillips-Beck, M Grisel Galarza, Maritza Torres, Sadaf Zindani, Ahmad Rayani, Khalee Edwards, Sande Gracia Jones, Jean Hannan

**Affiliations:** 1 School of Nursing University of Ottawa Ottawa, ON Canada; 2 Department of Communication Sciences and Disorders Florida International University Miami, FL United States; 3 Nicole Wertheim College of Nursing and Health Sciences Florida International University Miami, FL United States; 4 Chaplin School of Hospitality &Tourism Management Florida International University North Miami, FL United States; 5 Knight Foundation School of Computing and Information Sciences Florida International University Miami, FL United States; 6 IBM Miami, FL United States; 7 First Nations Health and Social Secretariat of Manitoba Winnipeg, MB Canada; 8 Miller School of Medicine, Pediatrics/Neonatology University of Miami Miami, FL United States; 9 College of Nursing King Saud University Riyadh Saudi Arabia

**Keywords:** maternal and child health, smartphone, mobile health, mHealth, eHealth, app development, app evaluation, app effectiveness, maternal and infant app, pregnancy, postpartum, mothers, mobile phone, artificial intelligence, AI

## Abstract

**Background:**

Globally, high rates of maternal and infant mortality call for interventions during the perinatal period to engage pregnant people as well as their loved ones in care. Mobile health technologies have become ubiquitous in our lives and in health care settings. However, there is a need to further explore their safety and effectiveness to support and improve health outcomes locally and globally.

**Objective:**

The aim of this study was to review and synthesize published literature that described the development process or effectiveness evaluations of maternal and infant apps.

**Methods:**

We applied a methodological framework for scoping reviews as well as the PRISMA-ScR (Preferred Reporting Items for Systematic Reviews and Meta-Analyses extension for Scoping Reviews) guidelines; in addition, the systematic review platform Covidence (Veritas Health Innovation Ltd) was used to facilitate the review of included studies. Search terms were developed collaboratively, and health sciences–associated databases were searched for studies conducted between January 1, 2000, and February 4, 2022. We excluded studies about apps that only gathered or tracked data or targeted care providers.

**Results:**

A total of 1027 articles were included for title and abstract screening, of which 87 (8.47%) were chosen for full-text screening. Of these 87 articles, 74 (85%) were excluded with reasons, and 19 (22%) were included. Four articles were added at data extraction from hand searching and 2 others were excluded. Thus, we reviewed and synthesized data from 11 unique studies reported in 21 articles published between 2017 and 2021. The included studies represented 8 different countries. Most of the apps (8/11, 73%) were in English, although apps were also developed in Arabic, Bahasa Indonesia, and Nepali. The articles reviewed revealed the early stage of development of the field of maternal and infant health apps, with modest evidence of app use and achievement of study outcomes. Only 1 (9%) of the 11 apps was endorsed by an independent health care provider society. App development and evaluation processes emerged, and specific app features were identified as vital for well-functioning apps. End-user engagement occurred in some, but not all, parts of app research and development.

**Conclusions:**

Apps to improve maternal and infant health are being developed and launched in enormous numbers, with many of them not developed with mothers’ needs in mind. There are concerns about privacy, safety, and the standardization of current apps as well as a need for professional or institution-specific guidelines or best practices. Despite challenges inherent in currently available apps and their design processes, maternal and infant app technology holds promise for achieving health equity goals and improving maternal and child health outcomes. Finally, we propose recommendations for advancing the knowledge base for maternal and infant apps.

## Introduction

### Overview

Achieving the global health goal of health for all requires engaging and empowering individuals, families, and communities for increased social participation and enhanced self-care and self-reliance in health, in addition to universal health coverage (UHC) and primary health care (PHC) [1-4]. Globally, high rates of maternal and infant mortality call for interventions during the perinatal period to engage pregnant people as well as their loved ones to ensure that they remain in care during pregnancy and the postpartum period [5-10]. As mobile health (mHealth) technologies such as smartphone apps emerge and become ubiquitous in our lives and in health care settings, there is a need to further explore their potential to support and improve health outcomes locally and globally. The COVID-19 pandemic demonstrated the capacity for widespread uptake of mHealth technologies in every aspect of life [11,12]. Before the COVID-19 pandemic, there were numerous smartphone apps being developed to support many diverse health goals [7-9,12-14]. However, many maternal and infant health apps are short lived or constrained to specific health care systems or networks, and few of them are evaluated for effectiveness in improving health outcomes for the mother, their children, and families or endorsed or reviewed by health professionals or organizations independent of app development teams [14-19]. Despite the existence of a plethora of apps to support parents, especially during the perinatal and postpartum periods, documented scientific data remain meager. The limited peer-reviewed published evidence about the development process and effectiveness of apps in supporting mothers or parents with the challenges they face during the perinatal or postpartum period makes the content of the available apps questionable, which may influence their efficacy.

### Background and Significance

#### Apps to Prevent Maternal and Infant Morbidity and Mortality

Numerous apps have been developed to support and improve maternal and infant health, including during pregnancy and the postpartum period. These apps can be an efficient means of providing information for parents, and the number of apps is rapidly increasing [20,21]. However, most apps lack the information needed and searched for by mothers with low income and non–English-speaking mothers with low income belonging to minority groups. It is well documented that people with low income, those with low income belonging to minority groups, and non–English-speaking people have a lower rate of pregnancy app use [22,23]. Most maternal and infant apps are not designed for women with low income and culturally diverse non–English-speaking women [24-26]. In the United States, it is estimated that most women (92%-95%) aged between 18 and 34 years own a smartphone [27]. This large proportion of smartphone users may have easy access to apps during pregnancy and the postpartum period when they could benefit from app-based maternal and infant health information. Evidence is emerging that maternal and infant apps have been developed and tested in resource-constrained settings and for use in humanitarian crises [7-9,14,28]. However, most existing pregnancy apps lack commercial regulation and standardization, making their content questionable [29]. Potential harm from several pregnancy mHealth intervention apps have been identified by health professionals [30]. Many apps have not been evaluated for content accuracy, making it difficult for users to assess the reliability of the information presented in them [31,32]. Many apps currently lack information that would be most helpful for women during pregnancy [33,34]. Neither medical nor health care societies have issued guidelines for mHealth apps [18,19,29,35,36]. Few studies exist that report on the outcomes from the use of such apps [29].

Regulatory agencies are constrained under current regulatory frameworks to provide effective and efficient regulation of apps that can be classified as software as medical device (SaMD) [17-19,35]. The US Food and Drug Administration (FDA) takes the position that the regulation of apps needs to be tailored to the risk and benefit profiles of the apps but has *no* standards for apps [35]. The FDA “oversees apps intended to treat, diagnose, cure, mitigate, or prevent diseases or other conditions as medical devices under federal statute” [35]. The FDA seeks to empower patients and clinicians through innovation, including the creation of regulatory frameworks that instills confidence in the performance and reliability of apps [35]. The International Organization for Standardization (ISO) has articulated assessment processes and quality requirements for health apps [17]. There are international standards for product safety and lifecycle processes that are applicable to health apps. However, because of the time investment involved, most health-related apps are not evaluated [17]. This lack of effective regulatory oversight has led to calls for user-centered reforms to improve the accuracy, usability, accessibility, and privacy protection features of apps, especially health apps [18,19].

The current research and regulatory landscape offers little data or regulatory guidance to inform people about the effectiveness of available apps that aim to improve health outcomes among mothers, especially mothers with low income, mothers with low income belonging to minority groups, and non–English-speaking mothers. The lack of regulatory frameworks and guidelines for the development of safe and effective maternal and infant apps limits the confidence of patients and clinicians and may lead to harms derived from the use of currently available apps [18,19,35,36]. Increasing knowledge in this area is important because the population of people with low income and those with low income belonging to non–English-speaking minority groups continues to grow, and these groups tend to have poorer maternal and infant health outcomes. In addition, there is an increased need for maternal and infant apps in languages other than English.

#### App Searches

Mobile apps are downloaded by end users on their smartphone. However, there are little data on why people search for apps, although major life events seem to be drivers for mobile app installations [37]. People experiencing major life events—change in marital status, moving, job change, pregnancy, or the birth of a child—install 2.5 times more apps than those without any significant life changes. There are studies reporting how end users find apps [37]. More than half of app users (55%) found apps based on recommendations from friends, family members, and colleagues [37]. In addition, 1 in 3 consumers found apps through app store recommendations; searching in an app store; and advertisements on the web, social media, and television. Most consumers (74%) downloaded apps after viewing mobile advertisements for them [38]. There are little data documenting that consumers’ app searches and downloads are based on scientific recommendations [38,39].

#### Brief Overview of Currently Available Parent and Infant Health Apps

An extensive review of currently available maternal and infant apps is beyond the scope of this review. In 2018, a total of 5276 Android maternal and child health (MCH) apps and 877 iOS MCH apps were identified [40,41]. There are estimated to be >350,000 health apps available worldwide, and it is estimated that 250 new health apps are released every daily [42].

### Positionality Statement

Our scoping review team includes professionals and researchers with a variety of perspectives that inform our evaluation of the literature reviewed. We represent multiple cultural backgrounds, migrant statuses, sexes, and genders. In addition, our multiple academic disciplines include computer technology and IT, communications, human rights law, informatics, speech-language pathology, medicine, and maternal and child nursing. We have team members from multiple contexts globally. Our varied lived experiences and knowledge support analysis of the literature reviewed from a wider perspective of world views to inform future development of computer-mediated technologies, such as smartphone apps, to improve the health of mothers, their infants, families, and communities.

### Objectives

The purpose of this scoping review study was to review and synthesize published literature that described the development process or effectiveness evaluations of maternal and infant health apps, with a specific emphasis on determining the use of the apps by the target population; provided evidence of outcomes with mothers, fathers, infants, or children; and explained whether the apps have been reviewed or endorsed by a health care provider. The research question guiding this scoping review study was as follows: what evidence exists that describes the development and effectiveness evaluation of maternal and infant health apps?

## Methods

### Scoping Review Approach

Because of the scarce evidence of apps being systematically evaluated for effectiveness, we used a scoping study methodology to review and synthesize the existing literature. The scoping review approach was originally described by Arksey and O’Malley [43] and has since been adapted by Islam et al [44], Levac et al [45], and Westphaln et al [46]. The original scoping review method included 5 steps: identifying the research question (step 1); search strategy (step 2); study selection (step 3); charting the data (step 4); and collating, summarizing, and reporting the results (step 5). Two additional steps were added subsequently: consultation (step 6) [45,46] and quality assessment (step 7) [44]. We used the PRISMA-ScR (Preferred Reporting Items for Systematic Reviews and Meta-Analyses extension for Scoping Reviews) guidelines to enhance transparency in our approach to our scoping study [47]. The PRISMA-ScR guidelines checklist is available in Multimedia Appendix 1.

### Steps Taken

The identification of the research question (step 1) and the development of our search strategy (step 2) were developed collaboratively during team meetings. The research question addressed by the scoping study was as follows: what evidence exists that describes the development and assessment of the development and effectiveness of parent and infant health apps? Specifically, we sought to identify extant studies that described the use of the apps by the target population; provided evidence of outcomes with mothers, infants, or children; and explained whether the apps have been reviewed or endorsed by a health care provider or health care provider society (eg, American Academy of Pediatrics). Our search strategy included literature published between January 1, 2000, and February 4, 2022. The search terms included “((mother* OR mom* OR matern* OR pregna* OR parent* OR postpart*) AND (infan* OR newborn OR neonat* OR prenat* OR perinat* OR postnat* OR bab*) AND (app OR mobile app OR apps OR mobile device applications OR mobile apps OR smartphone) AND (health*)).” The search resulted in 1895 citations being identified. The search process commenced on January 27, 2022, with a preliminary search of Academic Search Complete (EBSCO), Bibliography of Indigenous Peoples in North America (EBSCO), CINAHL, Communication Source (EBSCO), Education Source (EBSCO), and Global Health (EBSCO). The citations identified from this search (163/1895, 8.6%) were imported into the systematic review platform Covidence (Veritas Health Innovation Ltd) [48]. MEDLINE (Ovid) was also searched on January 27, 2022, and the citations identified (398/1895, 21%) were imported into Covidence [48]. Citations from Scopus (64/1895, 3.38%), PubMed (656/1895, 34.62%), and Web of Science (614/1895, 32.4%) were identified in an additional search on February 4, 2022, and added to Covidence [48]. Of the 1895 citations, after screening, 892 (47.07%) duplicates were removed.

Study selection (step 3); charting the data (step 4); and collating, summarizing, and reporting the results (step 5) were facilitated using Covidence [48]. Study selection occurred in 2 stages: title and abstract screening and full-text screening. All articles at each stage were reviewed by at least 2 team members. Any conflicts were resolved during team meetings for title and abstract screening. During full-text screening, any conflicts were resolved by team members who had differing opinions about inclusion discussing their differences and coming to an agreement about whether to include a citation for data extraction. Inclusion and exclusion criteria (Textbox 1) were specified during team meetings and adapted as needed through team consensus. All team members had the opportunity to participate in title and abstract screening, which aligns with our approach to consultation (step 6) that was inclusive of the multiple perspectives of our team members.

**Literature review inclusion and exclusion criteria.**

**Inclusion criteria**

- Published primary research article (eg, completed studies)
- Review article (eg, systematic review or scoping review)
- Apps for pregnant people (people), parents (include fathers if they are part of the app’s target audience), postpartum people (people), infants and children, and mothers and infants
- Language: app in any language; articles limited to publications in English
- Any country
- Article describes app development process or how effectiveness was determined (eg, randomized controlled trial or evaluation)

**Exclusion criteria**

- Study or app focused on pathology or psychopathology (eg, gestational diabetes mellitus, preterm or premature birth, anxiety, and depression)
- Study protocols
- Thesis or dissertation
- Commentaries, editorials, and letters to the editor
- Apps for health care or community services workers only
- Apps for data gathering or tracking
- Computer-mediated platforms: websites, communication platforms (eg, WhatsApp, Facebook Messenger, and FaceTime), and social media or social networking platforms (eg, Twitter, Facebook, and Reddit)

Our team developed a data extraction tool for charting the data (step 4). This instrument was then entered into Covidence to facilitate data extraction. Three authors (JCP, JH, and SZ) completed data extraction. All other team members had access to the data extraction outputs in Covidence [48]. The final outputs of the data extraction process—the charted data—were shared with all team members for review and discussion at a team meeting. Collating, summarizing, and reporting the results (step 5) were completed using the PRISMA-ScR process [47]. To ensure rigor in reporting our findings, we used a 3-stage process [45]. First, we provide numerical summaries of key aspects from the reviewed studies (eg, country where app was designed to be used, app language, and study population). Second, narrative summaries, tables, and figures are used to present our findings and facilitate comparisons between, and contrasts across, the reviewed studies. Finally, in the *Discussion* section, we elaborate on the implications of our findings for the future research and development of maternal and infant apps. We also propose recommendations for improving the development, usability, end-user uptake, evaluation, quality assessment, as well as policies for funders and regulators in the field.

Consultation (step 6) was incorporated into this scoping review by including the multiple personal and professional perspectives of the members of our diverse and inclusive team, which is briefly described in the *Positionality Statement* subsection. We did not consult outside our research team for conducting this scoping review study. Our future research endeavors will include wider community consultations to include the experiences and perspectives of the people who use maternal and infant apps.

Quality assessment (step 7) is a potentially fraught process for scoping review studies, but efforts are underway to develop an appraisal tool for them [49]. Some researchers have included this step to enhance scoping review quality [44]. For the purposes of our review and given the early developmental stages of the science regarding the development and effectiveness evaluations of smartphone apps, quality assessment was not part of the inclusion criteria for this study. The assessment of the selected studies will be made in a separate study after recommendations for the critical appraisal of scoping reviews have been more formalized [49].

## Results

### Overview

**Figure 1:**
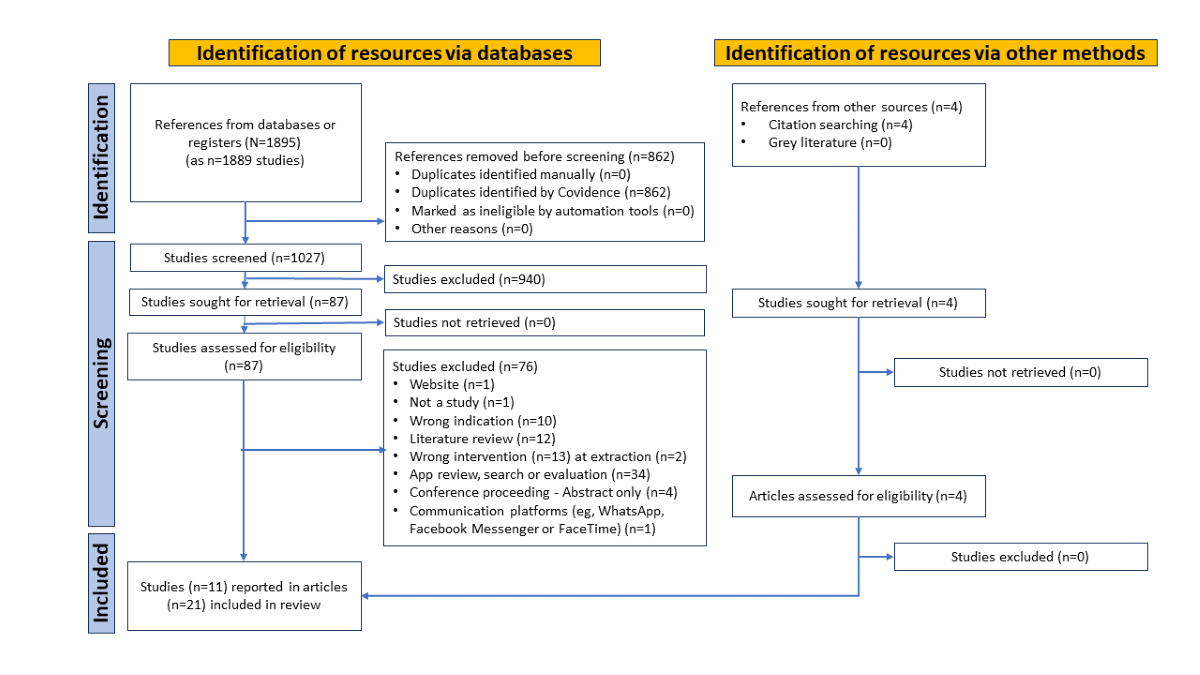
PRISMA (Preferred Reporting Items for Systematic Reviews and Meta-Analyses) flow diagram.

Of the 1889 studies identified, after removing 862 (45.63%) duplicates, 1027 (54.37%) articles remained. Of these 1027 articles, 940 (91.53%) were excluded during the title and abstract screening. Of the remaining 87 articles that were assessed for eligibility during full-text screening, 74 (85%) were excluded for reasons stated in the PRISMA (Preferred Reporting Items for Systematic Reviews and Meta-Analyses) diagram (Figure 1 [50]), resulting in 19 (22%) articles reporting on 13 distinct studies that were included for data extraction. At data extraction, 4 articles [51-54] describing aspects of 1 (8%) of these 13 studies were added from a hand search of the literature, yielding a total of 23 articles for data extraction. Of the total 23 articles, 2 (9%) were excluded at data extraction; 1 (4%) was excluded because the app is limited to podcasts, which may not offer a range of engagement opportunities and communication modalities for app users and has less potential for use with multiple languages [55]; and 1 (5%) was excluded because the study tested a model of care that included an encrypted digital app that facilitated text-based communication between patients and their care team, not an app with multiple functionalities [56]. Each of these excluded articles reported on a study, which yielded the final total of 11 studies reported in 21 articles included. Of these 11 studies, 2 (18%) were reported in multiple articles, 1 (9%) was reported in 3 (14%) of the 21 articles [7-9], and 2 (18%) studies were each reported in 5 (24%) of the 21 articles [51-54,57-62]. Ultimately, we reviewed and synthesized data from 11 unique studies reported in 21 articles, published between 2017 and 2021.

### Characteristics of Included Studies

Table 1 summarizes characteristics across the 11 included studies. The lead authors of the included studies represented 8 different countries, with Australia (3/11, 27% studies reported in 7/17, 41% of the articles) [57,59-64] and the United States (3/11, 27%) [16,65,66] having the greatest representation. The other represented countries included Indonesia (1/11, 9%) [28], Jordan (1/11, 9%) [14], Morocco (1/11, 9%) [67], Nepal (1/11, 9% study reported in 3/21, 14% of the articles) [7-9], and Singapore (1/11, 9% study reported in 5/21, 24% of the articles) [51-54,58]. The health discipline of the primary authors varied, with the most common being medicine (3/11, 27%) and nursing (3/11, 27%). The other disciplines included public health (2/11, 18%), followed by computer technology fields: computing and informatics (1/11, 9%), IT (1/11, 9%), and biomedical engineering (1/11, 9%). Most of the apps were in English (8/11, 73%); other app languages included Arabic (1/11, 9%) [14], Bahasa Indonesia (1/11, 9%) [28], and Nepali (1/11, 9% study reported in 3/21, 14% of the articles) [7-9]. English-language apps were developed for use in Australia (3/11, 27%), the United States (3/11, 27%), Morocco (1/11, 9%), and Singapore (1/11, 9%).

**Table 1:** Key characteristics of reviewed studies (n=11).

Country (language); authors and year			Studies (n=11), n (%)		Articles (n=21), n (%)		Disciplines		Endorsed by independent HCP^a^
**Australia (English)**			3 (27)		7 (33)				
	Dalton et al [64]; 2018					Anthropology, media, communications, and health (health, arts, and design)		No	
	Meedya et al [63]; 2021					Medicine, nursing, social work, IT, computer science, and business		No	
	Scott et al [57]; 2021					Medicine, nursing, IT, dietetics, public health, and population health		No	
	White et al [59]; 2016					Medicine, nursing, IT, dietetics, public health, and population health		No	
	White et al [60]; 2018					Medicine, nursing, IT, dietetics, public health, and population health		No	
	White et al [61]; 2016					Medicine, nursing, IT, dietetics, public health, and population health		No	
	White and Scott [62]; 2019					Medicine, nursing, IT, dietetics, public health, and population health		No	
**United States (English)**			3 (27)		3 (14)				
	Bush et al [65]; 2017					Nursing		No	
	Cawley et al [66]; 2020					Nursing, public health, and business administration		No	
	Chaudhry et al [16]; 2019					Medicine, social work, computer science, and trained health workers (prenatal care coordination providers)		No	
**Indonesia (Bahasa Indonesia)**			1 (9)		1 (5)				
	Wiweko et al [28]; 2019					Medicine and computer science		No	
**Jordan (Arabic)**			1 (9)		1 (5)				
	Nasir et al [14]; 2020					Medicine, international development agencies, UNRWA^b^, and World Bank		No	
**Morocco (English)**			1 (9)		1 (5)				
	Sardi et al [67]; 2020					Medicine, computer science, and biomedical science		Yes	
**Nepal (Nepali)**			1 (9)		3 (14)				
	Kayastha et al [7]; 2021					Social work, IT, and computer science (female community health volunteers were part of the sample studied)		No	
	Mueller et al [8]; 2020					Social work, IT, and computer science (female community health volunteers were part of the sample studied)		No	
	Mueller et al [9]; 2020					Social work, IT, and computer science (female community health volunteers were part of the sample studied)		No	
**Singapore (English)**			1 (9)		5 (24)				
	Shorey et al [58]; 2017					Nursing and psychiatry		No	
	Shorey and Ng [51]; 2019					Nursing and psychiatry		No	
	Shorey et al [52]; 2019					Nursing and psychiatry		No	
	Shorey et al [53]; 2021					Nursing and psychiatry		No	
	Shorey et al [54]; 2018					Nursing and psychiatry		No	

^a^HCP: health care provider.

^b^UNRWA: United Nations Relief and Works Agency for Palestine Refugees in the Near East.

The studies included a variety of study designs, including randomized controlled trial (2/11, 18% studies reported in 3/21, 14% of the articles) [51,57,58], observational study (1/11, 9%) [66], multisite cross-sectional study (1/11, 9%) [14], diagnostic test accuracy study (1/11, 9%) [16], mixed methods study (1/11, 9%) [63], case study methodology report of a pilot study (1/11, 9%) [65], retrospective review (1/11, 9%) [64], app development reports (2/11, 18%) [28,67], and qualitative articles with participants from the main study (4/11, 36%) [51,53,54,60]. Of the 11 apps, 4 (36%) were designed for use in resource-constrained settings: Indonesia [28], Morocco [67], Nepal (reported in 3/21, 14% of the articles) [7-9], and Palestine refugee camps in Jordan [14].

All studies reviewed reported that they had funding to conduct the research for the study. Of the 11 studies, 7 (64%) were funded by a governmental agency, whereas 1 (9%) was funded by a state Medicaid office [65], 1 (9%) was funded by the United Nations Relief and Works Agency for Palestine Refugees in the Near East (UNRWA) [14]; 1 (9%), reported in 5 (24%) of the 21 articles, was funded by a university [51-54,58]; and 1 (9%) was funded by a health system [66]. Funding specific for app development was reported in 5 (46%) of the 11 studies reported in 7 (33%) of the 21 articles [7-9,28,65-67]. Funding to support app sustainability was not specifically reported in any of the studies but could be assumed in 3 (27%) of the 11 studies [14,16,65]. It was not clearly specified whether app development and sustainability funding were obtained for 2 (18%) of the 11 studies [14,16].

### Evidence of Apps’ Use, Outcomes, or Endorsement

Characteristics of the study populations from the reviewed studies are summarized in Table 2, and evidence use of the apps by the target population is presented in Table 3. Sardi et al [67] described an app in development and proposed a study to evaluate the effectiveness of the app they developed in collaboration with postpartum people. Evidence of outcomes with mothers, fathers, infants, and children was limited and is summarized in Table 3. Evidence that apps have been reviewed or endorsed by a health care provider is presented in Table 1. Although all studies reviewed included health professionals or health care providers as members of their research and development teams, only 1 (9%) of the 11 apps was endorsed by an independent health care provider or health care provider society not involved in the app’s development or evaluation [67].

**Table 2:** Participant characteristics.

Authors	Population description	Recruitment method	Sample size, n	Sample characteristics
Sardi et al [67]	Physicians and nurses (app for puerperal women)	Hospital	NR^a^	NR
Wiweko et al [28]	Pregnant and nonpregnant people	Clinic patients	205	NR
Chaudhry et al [16]	Prenatal care coordination providers, social workers, and women	Clinic patients	9	Age: 20-36 y Ethnicity: African American (6/9, 67%); Hispanic (1/9, 11%); White (2/9, 22%) Education: ≤high school (6/9, 67%); college (3/9, 33%) Income: US $0-US $30,000/y
Meedya et al [63]	Pregnant people	News platform, paper flyers, and social media	7	Age: 29-37 y Race or ethnicity: Asian; European; Middle Eastern; White Education: NR Income: >US $6000/mo
Bush et al [65]	Pregnant people	Grass roots referrals	85	NR
Shorey et al [58]	Couples (mothers and fathers)	Clinic patients	250 (126/250, 50% [63 couples] received education support via app, whereas 124/250, 50% [62 couples] were in the control group)	Age: 26-42 y Ethnicity: Chinese; Malay; other Education: NR Income: >SG $6000 (US $4367)/mo
Shorey and Ng [51]	Couples (mothers and fathers)	Clinic patients	250 (126/250, 50% [63 couples] received education support via app, whereas 124/250, 50% [50] couples were in the control group)	Age: 26-42 y Ethnicity: Chinese; Malay; other Education: NR Income: >SG $6000 (US $4367)/mo
Shorey et al [52]	Couples (mothers and fathers)	Clinic patients	250 (126/250, 50% [63 couples] received education support via app, whereas 124/250, 50% [50] couples were in the control group)	Age: 26-42 y Ethnicity: Chinese; Malay; other Education: NR Income: >SG $6000 (US $4367)/mo
Shorey et al [53]	Couples (mothers and fathers)	Clinic patients	250 (126/250, 50% [63 couples] received education support via app, whereas 124/250, 50% [62 couples] were in the control group)	Age: 26-42 y Ethnicity: Chinese; Malay; other Education: NR Income: >SG $6000 (US $4367)/mo
Shorey et al [54]	Couples (mothers and fathers)	Clinic patients	250 (126/250, 50% [63 couples] received education support via app, whereas 124/250, 50% [62 couples] were in the control group)	Age: 26-42 y Ethnicity: Chinese; Malay; other Education: NR Income: >SG $6000 (US $4367)/mo
Nasir et al [14]	Parents (mothers and fathers)	Clinic patients	1042	Age Mothers: 23-33 y Fathers: 29-39 y Ethnicity: Palestinian (refugees) Education: NR Income: US $0
Cawley et al [66]	Postpartum mothers	Mail	567	Age: 20-36 y Race or ethnicity: Asian (74/567, 13%); Hispanic (46/567, 8%); White (360/567, 63%); other (87/567, 15%) Education: ≤high school (82/567, 14%); college (482/567, 85%) Income: US $0-US $70,000/y (276/567, 49%); >US $70,000-US $150,000/y (201/567, 35%)
Scott et al [57]	Expecting couples (mothers and fathers)	Clinic patients	1426	Age Mothers: 33-34 y Fathers: NR Race or ethnicity: African or Middle Eastern (64/1426, 4%); Asian (84/1426, 6%); Australia or New Zealand (724/1426, 51%); United Kingdom or Ireland (129/1426, 9%); other (72/1426, 5%) Education: ≤high school (409/1426, 29%); college (663/1426, 46%) Income: NR
White et al [59]	Expecting couples (mothers and fathers)	Clinic patients	1426	Age Mothers: 33-34 y Fathers: NR Race or ethnicity: African or Middle Eastern (64/1426, 4%); Asian (84/1426, 6%); Australia or New Zealand (724/1426, 51%); United Kingdom or Ireland (129/1426, 9%); other (72/1426, 5%) Education: ≤high school (409/1426, 29%); college (663/1426, 46%) Income: NR
White et al [60]	Expecting couples (mothers and fathers)	Clinic patients	1426	Age Mothers: 33-34 y Fathers: NR Race or ethnicity: African or Middle Eastern (64/1426, 4%); Asian (84/1426, 6%); Australia or New Zealand (724/1426, 51%); United Kingdom or Ireland (129/1426, 9%); other (72/1426, 5%) Education: ≤high school (409/1426, 29%); college (663/1426, 46%) Income: NR
White et al [61]	Expecting couples (mothers and fathers)	Clinic patients	1426	Age Mothers: 33-34 y Fathers: NR Race or ethnicity: African or Middle Eastern (64/1426, 4%); Asian (84/1426, 6%); Australia or New Zealand (724/1426, 51%); United Kingdom or Ireland (129/1426, 9%); other (72/1426, 5%) Education: ≤high school (409/1426, 29%); college (663/1426, 46%) Income: NR
White and Scott [62]	Expecting couples (mothers and fathers)	Clinic patients	1426	Age Mothers: 33-34 y Fathers: NR Race or ethnicity: African or Middle Eastern (64/1426, 4%); Asian (84/1426, 6%); Australia or New Zealand (724/1426, 51%); United Kingdom or Ireland (129/1426, 9%); other (72/1426, 5%) Education: ≤high school (409/1426, 29%); college (663/1426, 46%) Income: NR
Kayastha et al [7]	Men and women	By referrals	71	NR
Mueller et al [8]	Men and women	By referrals	71	NR
Mueller et al [9]	Men and women	By referrals	71	NR
Dalton [64]	Pregnant people	Clinic patients	124	Age: 19-41 y Ethnicity: Australian White (103/124, 83%); other (21/124, 17%) Education: ≤high school (83/124, 67%); college (41/124, 33%) Income: NR

^a^NR: not reported.

**Table 3:** Target group involvement, app use, and outcomes.

Authors	Target group	Involvement	App use	Outcomes reported
Sardi et al [67]	Mothers and infants	App development	N/A^a^ (app in development phase)	Clinical staff examined app features and functionalities. A future study with mothers is planned.
Wiweko et al [28]	Mothers	Implementation phase	Mothers	App provides pregnant people directions to nearest health centers, access to medical staff, and saves patient’s medical records to easily obtain professional help needed immediately.
Chaudhry et al [16]	Mothers and infants	App development	Pregnant people	Low use by both providers and mothers.
Meedya et al [63]	Mothers	App development	Breastfeeding mothers	App was piloted with, and revised based on, mothers’ feedback.
Bush et al [65]	Pregnant people	NR^b^	Pregnant people	There was a statistically significant increase in the completion of prenatal visits (*P*=.02). There was an association between the use of the app and lowered incidence of low birth weight infants (*P*=.06).
Shorey et al [58]	Postnatal mothers and fathers	Research process	Postnatal mothers and fathers	There was an increase in the parenting confidence of new parents, better perceived social support (parents were encouraged to proactively seek help), and greater parenting satisfaction.
Shorey and Ng [51]	Postnatal mothers and fathers	Research process	Postnatal mothers and fathers	There was an increase in the parenting confidence of new parents, better perceived social support (parents were encouraged to proactively seek help), and greater parenting satisfaction.
Shorey et al [52]	Postnatal mothers and fathers	Research process	Postnatal mothers and fathers	There was an increase in the parenting confidence of new parents, better perceived social support (parents were encouraged to proactively seek help), and greater parenting satisfaction.
Shorey et al [53]	Postnatal mothers and fathers	Research process	Postnatal mothers and fathers	There was an increase in the parenting confidence of new parents, better perceived social support (parents were encouraged to proactively seek help), and greater parenting satisfaction.
Shorey et al [54]	Postnatal mothers and fathers	Research process	Postnatal mothers and fathers	There was an increase in the parenting confidence of new parents, better perceived social support (parents were encouraged to proactively seek help), and greater parenting satisfaction.
Nasir et al [14]	Pregnant people and mothers	No community involvement	Pregnant people and mothers	The number of participants who downloaded the app was reported. Having other apps (OR^c^ 6.17; *P*<.01), staff knowledge of the app (OR 11.82; *P*<.01), using the Internet as a source of medical information (OR 1.63; *P*=.01) and having internet access at home (OR 1.46; *P*=.05) were associated with app download.
Cawley et al [66]	Mothers and infants	Research process	Pregnant people	The app provided access to personalized and evidence-based health information. The app was associated with an increase in healthy behaviors and health knowledge.
Scott et al [57]	Mothers and fathers	Research process	Fathers	The study did not demonstrate a measurable impact of father-focused support for breastfeeding.
White et al [59]	Mothers and fathers	Research process	Fathers	The study did not demonstrate a measurable impact of father-focused support for breastfeeding.
White et al [60]	Mothers and fathers	Research process	Fathers	The study did not demonstrate a measurable impact of father-focused support for breastfeeding.
White et al [61]	Mothers and fathers	Research process	Fathers	The study did not demonstrate a measurable impact of father-focused support for breastfeeding.
White and Scott [62]	Mothers and fathers	Research process	Fathers	The study did not demonstrate a measurable impact of father-focused support for breastfeeding.
Kayastha et al [7]	Pregnant people and mothers	App development	Pregnant people and mothers	Participants gained more knowledge on maternal health than on neonatal health.
Mueller et al [8]	Pregnant people and mothers	App development	Pregnant people and mothers	Participants gained more knowledge on maternal health than on neonatal health.
Mueller et al [9]	Pregnant people and mothers	App development	Pregnant people and mothers	Participants gained more knowledge on maternal health than on neonatal health.
Dalton et al [64]	Mothers	App development	Pregnant people	There was a high rate of noncompletion among study participants.

^a^N/A: not applicable.

^b^NR: not reported.

^c^OR: odds ratio.

### App Development and Evaluation Processes

The studies reviewed revealed several approaches to app development. Some of the studies (4/11, 36%) used systematized app development strategies, including software requirements specification [67], rapid iterative testing and evaluation [16,57], and persuasive system design model and principles [63]. Most of the studies (9/11, 82%) included formalized evaluation processes. Standardized approaches to the evaluation of the apps included the Computer System Usability Scale [16], the Mobile Application Rating Scale [57,59,61], and investigator-developed evaluation instruments or processes [7-9,14,59].

### App Features

Each app included features intended to improve the end users’ experience. A full list of app features described in the studies is beyond the scope and purpose of this scoping review report. Textbox 2 summarizes the key features and functionality reported across the reviewed studies.

**Summary of the key app features and functionality reported across the reviewed studies.**

**Feature and functionality**

- Health status tracking: mechanism to record various health indicators and observe changes over time
- Care support and access to information: provides information to guide care and increase knowledge
- Usability: enhances the app user’s experience
- Health data protection and privacy: protects the end users’ health data gathered by, or shared through, the app
- Data transfer: allows for the sharing of information between patients and providers
- Communication with health care providers: facilitates dialogue and communication between patients and providers
- Behavior change techniques: mechanisms to change health-promoting or risk behaviors

### End-User Engagement

End-user engagement in app development was reported in 5 (45%) of the 11 studies, which were reported in 8 (38%) of the 21 articles [7-9,16,28,63,64,67]. Mothers were involved in app development in 7 (64%) of the 11 studies, which were reported in 4 (19%) of the 21 articles [16,28,63,67]. Fathers were involved in app development in 1 (9%) of the 11 studies, which was reported in 3 (14%) of the 21 articles [7-9]. End users were engaged in the research process in 3 (27%) of the 11 studies, which were reported in 11 (52%) of the 21 articles [51-54,57-62,66]. Of the 11 studies, 2 (18%) included mothers and fathers in the research process, as reported in 10 (48%) of the 21 articles [51-54,57-62]; and 1 (9%) included postpartum mothers in the research process [66]. Of the 11 studies, 1 (9%) included pregnant and nonpregnant people in the implementation phase of app development [28], whereas 2 (18%) did not report including end users in any aspect of the study [14,65].

## Discussion

### Principal Findings

#### Overview

Our scoping review is consistent with what has been previously reported in the literature. Apps have been developed for, and used in, a variety of settings globally. There are little data and regulatory guidance to inform people about the effectiveness of available apps that aim to improve health outcomes among mothers, especially mothers with low income, mothers with low income belonging to minority groups, and non–English-speaking mothers. This includes geographic locations with constrained resources and humanitarian crises (both human-made and natural disasters) [7-9,14,28]. The studies we reviewed reinforce the importance and usefulness of maternal and infant health apps to support global PHC objectives and confirm that they can be useful tools to facilitate the achievement of UHC [1-4]. However, our findings highlight several research gaps and challenges for the effective and sustainable development, implementation, and evaluation of maternal and infant health apps.

#### App Development Process

Currently, the development of maternal and infant health apps (including for use during pregnancy and the postpartum period) is on the rise; however, as documented in the literature and the results of this scoping review study, evaluation is lacking. Consistent with previous research, these apps are an efficient means of providing a wide range of health and safety information, and most women and parents, regardless of background or language, own a smartphone [7-9,14,20,21,28]. In fact, >85% of the world’s population in advanced economies [68] and >67% of the global population own a smartphone, with >90% owning a mobile phone [69,70]. Smartphone ownership makes health information on pregnancy and perinatal periods easily accessible through maternal and infant health apps. However, as seen in our study, maternal and infant health apps lack commercial regulation and standardization, making their content questionable, which has been previously documented [29]. As there is a lack of regulation and standardization, potential harm has been identified by health professionals with several pregnancy mHealth intervention apps [18,19,30,35]. Our review as well as other studies have found that many apps have not been evaluated for content accuracy, making it difficult for end users to assess the reliability of the information presented in them [31,32]. Some apps also lack information that would be most helpful for women and their families during the perinatal period [33,34]. No medical society has issued guidelines for mHealth apps [29], although the ISO and FDA offer guidance to support further development of guidelines [17,35], and legal scholars have proposed a framework for user-centered approaches to improve the safety and security of all apps, including mHealth apps [18,19].

In this scoping review study, we found that the outcomes reported demonstrated slight increases in behavior and knowledge [9,52,58,63,65,66], whereas other studies reported low use [14,16,64] or were in the development stages with no outcomes reported [28,67]. This is similar to other studies reporting on outcomes regarding the reasons why most apps developed are targeted at English-speaking White women without regard for women of other cultures and non–English-speaking people [8,14,22,23,28,29]. This has been attributed to a lack of app development designed for culturally diverse non–English-speaking women [25,71]. Few studies with culturally diverse women with low income and their use of mHealth apps have been reported or have examined language and cultural issues as potential barriers to app use [8,14,72,73]. Our study indicated that most of the apps (8/11, 73%) were in English. However, our scoping review study documents emerging evidence to support the use of maternal and infant health apps in other languages and cultures [7-9,14,28,51-54,58,67]. Studies have reported high uptake and use of linguistically and culturally tailored apps [74,75].

The findings of our study help in assessing similar conclusions in other recent studies that women using maternal and infant health apps during pregnancy and the postpartum period prefer greater and immediate access to information that is relevant to their local health care context, which includes support offered by health care professionals [25,76].

#### App Features

A summary of key features to include in future apps are described in Textbox 2. Key features for inclusion in apps include health status tracking, care support and access to information, usability, health data and privacy protection, data transfer, communication with health care providers, and behavior change techniques. Health status tracking facilitates recording various health indicators that can be monitored over time. Care support and access to information build knowledge to improve health outcomes. Usability enhances the end users’ experience when using an app. Health data and privacy protection protects the end users’ health data gathered by, or shared through, the app. Data transfer allows for sharing information between patients and health care or social services providers. Communication with health care providers facilitates dialogue and communication between patients and health care or social services providers. Behavior change techniques can be embedded in apps to support the achievement of health-promoting or risk behavior reduction goals. Additional information regarding app features is provided in a literature review conducted by Sardi et al [77].

In resource-constrained settings, such as Nepal [7-9], the app served multiple purposes to achieve public health and safety objectives, including maternal health and disaster preparedness. In addition, in refugee settings, an app based on the *Maternal and Child Health Handbook* contains basic MCH information and promotes care-seeking behaviors, improves the continuum of care, and increases users’ health-related behaviors [14]. This is evidence that apps can serve multiple health-related objectives, which has been documented in other settings during the COVID-19 pandemic [11,12]. In the context of health and humanitarian crises, the adoption of mHealth apps may be a wise use of scarce resources to address multiple public health–related and safety objectives simultaneously.

Potential risks related to mHealth and privacy exist and have been documented in the literature; for example, apps with the capacity to gather and store health data from end users need to have policies and protocols in place to ensure that the privacy of these data is maintained. These policies and protocols need to be transparent so that end users can be aware of who has access to their health data and for what purposes. In addition, algorithms, artificial intelligence, and machine learning can be used with the data gathered from apps. People who use these apps need to be aware of how these technologies are used with the data they share in apps [36]. Finally, risks can occur related to end users’ capability and capacity to read and understand content embedded in apps, even if the app is developed in the end users’ native language.

#### End-User Engagement

A fundamental feature of PHC that effective maternal and infant mHealth apps can offer is engaging people in their health care through empowerment and opportunities for enhanced self-care and self-reliance [1-4]. End-user engagement ought to be an essential part of the development of all maternal and infant health apps as well as other mHealth apps. Including end users in all stages of app development, implementation, scale-up, evaluation, and research across all stages is critical to the sustainability of apps and may enhance app longevity. Strategies for how to engage end users of apps in research have been described previously [78]. None of the studies included in this scoping review included participants in all aspects of app research and development. Most of the studies (9/11, 82%) included end users in part of the app research and development process, including app development, reported in 7 (33%) of the 21 articles [7-9,16,63,64,67]; the implementation of the app [28]; and the research process, reported in 11 (52%) of the 21 articles [51-54,57-62,66].

#### Quality Appraisal and Risk of Bias

The current state of the science for app development and evaluation limits the ability to evaluate the published studies for risk of bias [49]. Furthermore, there is debate about whether and how to review study quality and risk of bias in scoping reviews [49,79]. As our scoping review included a variety of different research approaches or app development reports, it was difficult to conduct a thorough quality appraisal of the potential for risk of bias, especially because we did not exclude any study based on quality appraisal or risk of bias. Our finding that the current literature may not meet criteria specified in many quality appraisal and risk-of-bias tools aligns with the challenges in the field of mHealth app development and evaluation with which regulatory and standards agencies are currently grappling [18,19,35].

### Strengths and Limitations of the Review

This scoping study used a methodological approach that has demonstrated success in other settings. In addition, we used the PRISMA-ScR guidelines to guide our study, which increases the transparency of the processes used to conduct the study. The limitations of this review include the fact that we may have missed some studies by only searching English-language literature. As we excluded studies with a primary focus on mental health outcomes, we may have missed some studies that reported on apps that have demonstrated efficacy and have begun to surmount the concerns with regard to quality and reliability as well as the accuracy, usability, accessibility, and privacy protection features of apps [18,19,35].

### Conclusions

In conclusion, this is one of the few studies reviewing the research regarding apps for maternal and infant health. These apps are increasingly being developed and launched in the marketplace in enormous numbers with little to no evaluation criteria in place. Many of the current maternal and infant health apps being launched are not developed with the pregnant person or mother’s needs in mind. Although the use of maternal and infant apps in health research is a relatively new area, there are concerns about the safety of these apps for end users. Future initiatives are needed to support health researchers to navigate the landscape of maternal and infant health apps and evaluate the impact of their efforts to develop effective and sustainable apps. Given the concerns related to safety and standardization, future research needs to focus on providing additional direction to health researchers on how to set policies in place. This could include the development of professional or institution-specific guidelines or the development of best practices. Furthermore, there is a need for research to determine the influence and implications of the integration of apps within health care information systems. The integration of apps into health care information systems architecture and environments may pose unique challenges that directly influence the acceptability and usability of these apps for end users and may limit an app’s utility, uptake, and sustainability. Despite challenges inherent in currently available apps and their design processes, maternal and infant health app technology holds promise for achieving health equity goals and improving MCH outcomes.

### Recommendations

Funders should consider strategies to support the sustainability of effective apps that achieve their stated purpose and are accessible, acceptable, safe, and secure for their end users. This will facilitate the sustainability of apps that have demonstrated effectiveness among pregnant people, parents, and their families. This implies that a quality appraisal or effectiveness evaluation of apps would need to be built into the app development, implementation, and scale-up processes.

We advocate for regulation to ensure that maternal and infant apps support the needs of mothers, fathers, and others who use them to improve health outcomes for mothers, infants, and their families. The regulatory framework proposed by Knox and Tenenbaum [18,19] would be useful to inform and guide regulatory advances in the field, as would the inclusion of strategies to protect the private information of people who use apps [18,19,36]. One aspect of this recommendation is for funders and policy makers to consider requiring end-user engagement in all aspects of app development and research that is consistent with the principles of PHC and UHC [1-4].

Researchers, policy makers, and patient advocates should advocate for the safe and wise use of new technology advances such as the artificial intelligence chatbots ChatGPT and Bard. These technologies may further advance opportunities for computer-mediated approaches that support improvements in MCH. These technologies hold tremendous potential to revolutionize health care but must be used to support goals for improved health outcomes, not for nefarious purposes.

## Appendix

Multimedia Appendix 1 PRISMA-ScR (Preferred Reporting Items for Systematic Reviews and Meta-Analyses extension for Scoping Reviews) checklist.

## Abbreviations

FDA: Food and Drug Administration
ISO: International Organization for Standardization
MCH: maternal and child health
mHealth: mobile health
PHC: primary health care
PRISMA: Preferred Reporting Items for Systematic Reviews and Meta-Analyses
PRISMA-ScR: Preferred Reporting Items for Systematic Reviews and Meta-Analyses extension for Scoping Reviews
SaMD: software as medical device
UHC: universal health coverage
UNRWA: United Nations Relief and Works Agency for Palestine Refugees in the Near East

## Data Availability

All data analyzed in this study are cited in this paper and available in the public domain. Data extraction tables are available from the corresponding author.
